# The impact of interactive book sharing on child cognitive and socio-cognitive development (the REaL trial): study protocol for a randomized controlled trial

**DOI:** 10.1186/s13063-022-06733-8

**Published:** 2022-09-24

**Authors:** Linda Forssman, Janna M. Gottwald

**Affiliations:** Department of Psychology, Uppsala University, von Kraemers allé 1, 75142 Uppsala, Sweden

**Keywords:** Cognitive development, Socio-cognitive development, Language development, Infant, Executive function, Parent intervention, Book sharing, Dialogic reading

## Abstract

**Background:**

The quality of children’s early home learning environment has an influence on their cognitive development, preliteracy skills, and subsequent educational outcomes. Early intervention programs that promote positive parenting behaviors and child cognition have great potential to positively influence children’s school readiness and thereby support social equality. One often advocated parental practice for promoting child language and cognition is interactive book sharing.

**Methods:**

We have conducted a randomized controlled trial to evaluate the effects of a parent-child interactive book sharing intervention on early child language, cognition, and parental behaviors. Participating caregivers and their 10-month-old child were randomized to an interactive book sharing intervention group (*n* = 59) or to an active control group (*n* = 56). The intervention was delivered by a facilitator to small groups of parent-child dyads on a weekly basis over 5 weeks. The primary outcomes were child language and socio-cognition; secondary outcomes were child executive function and parental scaffolding, sensitivity and reciprocity during book sharing, and problem-solving tasks. Data were collected at baseline, post-intervention, and at 6 and 12 months post-intervention.

**Discussion:**

*The Roadmap to Executive function and Language* (REaL) trial aims to evaluate the impact of a brief early parenting intervention on key factors for child development, including child cognition and parental behaviors. If this intervention is beneficial for child outcomes, that would be of significance for the development of early interventions to promote child development.

**Trial registration:**

The REaL trial is registered on the International Standard Randomized Controlled Trial Number database, registration number ISRCTN22319305. Retrospectively registered on 7 February 2020.

**Supplementary Information:**

The online version contains supplementary material available at 10.1186/s13063-022-06733-8.

## Background

Children’s early language and cognitive development are important precursors of their literacy skills and subsequent school progress [1–4]. The quality of children’s home learning environment is critical in shaping these developmental outcomes [5–7]. Children who grow up in high-quality home learning environments (e.g., with high availability of literacy resources, shared-reading experiences, and stimulating verbal engagements) tend to be set on a superior learning trajectory, compared to their peers from less advantageous environments [8, 9]. Without preventative strategies, individual differences in learning experiences and cognitive development during the early years can become magnified when children begin formal schooling [10, 11] and continue to influence their academic achievement throughout the school years [6, 12]. It has been suggested that early parenting interventions present a preventive strategy with a high potential to reduce these differences [13, 14].

One often advocated parental practice for promoting child cognition and literacy development is the sharing of picture- and storybooks. Shared book reading aids the establishment of joint attention between the child and their parent (or other social partner), which is a context believed to support language development [15–18]. In comparison with other activities, such as play and meal time, book sharing has been found to be more stimulating for children’s language development [16, 19–21]. Shared reading is associated with children’s vocabulary gains, emergent literacy, reading development (e.g., [22, 23]), and narrative development (e.g.,[24, 25]).

Strong evidence for the value of shared book reading for child development comes from intervention studies aimed at improving the quality of book sharing. Many of these intervention studies have assessed the effect of training parents in “*interactive reading*” (or “dialogic reading”) methods [26–28]; see also [29] for a review on shared reading intervention studies focusing on children with developmental disability/delay]. These methods, first described by Whitehurst and colleagues (e.g., [30–32]), generally build on Vygotskian principles [33], in which the parent scaffolds their child’s learning experience by supporting and responding to the child’s interests and cues and by providing stimulation. What differentiate these methods from “reading-as-usual” is the use of evocative techniques (i.e., actively engaging the child during reading), the provision of feedback (e.g., giving praise, using expansions and decontextualization), and the progressive change (i.e., adapting the shared reading style to the child’s developmental level).

Two early meta-analysis, on the added value of using interactive book sharing in contrast to reading-as-usual [27] and the effect of frequent parent-child book sharing [22], reported substantial benefits to child vocabulary development. The meta-analysis by Mol et al. [27] also indicated that younger children (2- to 3-year-olds) benefitted much more from interactive reading interventions than relatively older children (4- to 5-years-olds). Thus, interactive reading interventions for caregivers of young children could be an efficient mean of stimulating early language growth. Yet, few previous interactive reading interventions have targeted infants (but see [34]).

Two recent meta-analysis [26, 28] have assessed the effect of parental interactive book sharing on 1- to 6-year-old children’s language gain in randomized controlled studies (RCT) studies. Both studies corroborate the conclusion of the efficacy of interactive book sharing for children’s vocabulary development. The study by Dowdall et al. [26] reported small sized effects on expressive (*d* = .41, CI = .20, .61) and receptive (*d* = .26, CI = .12, .40) vocabulary, but larger effects were found for studies with medium to high intervention dose (i.e., more than 60 min contact time). This meta-analysis also showed a large sized intervention effects on caregiver book sharing competence (*d* = 1.01, CI = .40, 1.63), such as “caregiver expansion during book sharing,” “parental sensitivity and reciprocity during book sharing,” and “caregiver use of an interactive reading style.” However, one important caveat is that only five of the 19 studies included in the meta-analysis provided such data and these studies were limited due to small sample sizes and/or absence of an active control group. It was further reported that group-based interventions were associated with greater intervention gains in language compared to one-on-one interventions. Although speculative, the latter finding could indicate that the group-based format offered more learning opportunities through participant sharing their experiences or through increased social support [26]. Another possibility is that the group format increased participant adherence to the intervention protocol. This finding also points to the importance of using an adequately designed active control group instead of a no-contact (wait-list) group to control for nonspecific intervention effects, such as effects of increased social support in group interventions.

To date, there is little evidence regarding the effects of interactive book sharing on typically developing children beyond the language domain. Yet, it seems very plausible that book sharing also promotes child socio-cognition, specifically the ability to share attention (i.e., joint attention), since the book sharing context provides a structure that facilitates long episodes of shared attentional focus and communication. Furthermore, book sharing training programs that involve guidance for parents in how to best scaffold their child’s learning experience could also be of considerable benefit for children’s nonsocial cognitive development. This might be particularly true for child executive functions (EF), given the robust associations between parental scaffolding and EF development (e.g., [35, 36]). Besides the scarcity of reports on intervention effects beyond the language domain, there is also a need for a better understanding of the mechanisms responsible for intervention effects in studies on interactive book sharing. One previous RCT study for parents with 14- to 16-month-old children revealed that intervention gains in parental sensitivity and reciprocity mediated child language and attention gains [37]. However, this study is a notable exception and the reporting of parental competence and intervention fidelity is rare [26, 38]. Further, few studies have examined outcomes beyond the immediate post intervention (but see [34] for one exception). Thus, there is a paucity of intervention studies that has researched the endurance of the effects or potential long-term spill-over effects.

### Objectives

This RCT study, the REaL-trial (ISRCTN22319305), was designed to evaluate the effect of a 5-week book sharing intervention for caregivers of 10-month-old children. Parent-child dyads were randomly allocated to an intervention group or an active control group. The specific intervention involved teaching different interactive scaffolding techniques for caregivers to apply during book sharing with their child. These scaffolding techniques were child-centered and highlighted the importance of following the child’s lead, such as emphasizing stimuli to which child attends, using repetition, linking the book’s content to the child’s world, and helping the child to stay on task [37, 39, 40]. We collected data on child language, socio-cognition, and EF at baseline, post-intervention and at two follow-ups (6- and 12-months post-intervention). Assessments of caregiver competence were conducted at baseline and post-intervention.

### Hypothesis

Primary hypotheses:

(i) The intervention group will evidence significantly better outcomes on measures of language and socio-cognition (i.e., joint attention) at the post-intervention compared to the active control group and (ii) intervention gains in language and joint attention will predict better language development at the 18-month and 24-month follow-ups.
Performance on behavioral language measure at baseline and post-intervention will be correlated with a neurophysiological language measure (i.e., the event-related brain potentials component N400). We specifically expect a correlation between intervention gains and an increase in the N400 at the post-intervention.

Secondary hypotheses:

3. We predict long-term intervention effects on measures of language (expressive and receptive) and joint attention and spill-over effects on child EF. Thus, we expect that compared to the control-group children, intervention-group children will evidence significantly better on the specified outcome measures at 18 and 24 months of age.
4. We hypothesize that the intervention will improve parental scaffolding, sensitivity, and reciprocity and that these improvements mediate long-term child outcomes (language, joint attention and EF) at the follow-up assessments

## Methods: participants, interventions, and outcomes

### Study design

**Fig. 1:**
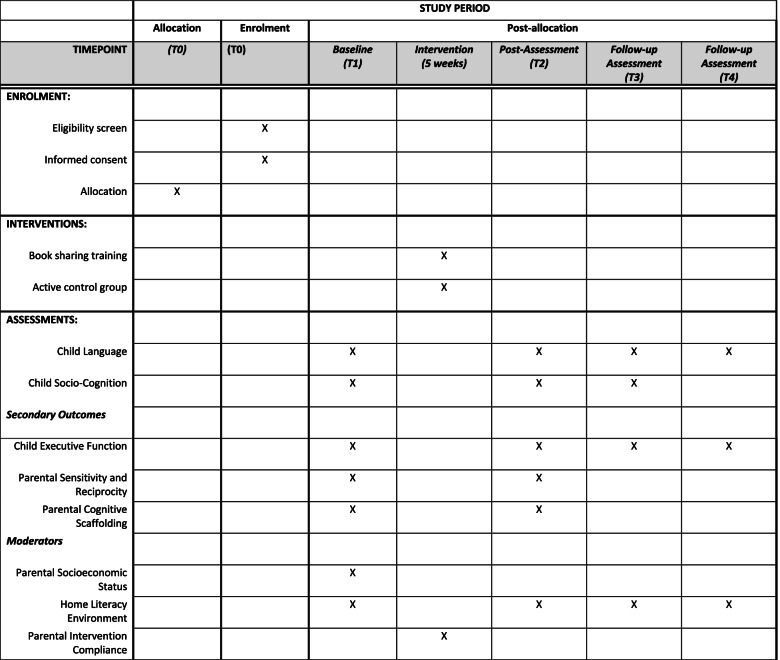
Schedule of enrolment, interventions, and assessments. Standard Protocol Items: Recommendations for Intervention Trials (SPIRIT) figure displaying schedule of enrolment, interventions, and assessments

The study is a two-arm RCT. In *the index condition*, caregivers received training in interactive shared book reading. In *the active control condition*, caregivers received general information about child development and played together. Randomization was performed as block randomization with a 1:1 allocation. Data collection takes place at four time points: (1) baseline prior to the start of the intervention, (2) at post-assessment, immediately following the intervention, (3) 6 months post-intervention, and (4) 12 months following post-assessment. The trial will run from 2020 to 2022. Figure 1 presents a Standard Protocol Items: Recommendations for Interventional Trials (SPIRIT) figure displaying the schedule of enrolment, interventions, and assessments. The SPIRIT guidelines for study protocols are followed and a SPIRIT checklist included (see Additional file 1).

### Study setting

The study is being conducted in Uppsala, a university town in Sweden (population size approximately 165,000). All lab assessments took place at the Uppsala Child & Baby Lab at Uppsala University. Intervention sessions were held at “open pre-schools” (a free of charge organization that caregivers can attend with their child and meet other caregivers and children), at libraries, and at the Uppsala University campus.

### Eligibility criteria

Prior to enrollment, caregivers who had received information about the study and were interested in participating in the study with their child were screened for inclusion and exclusion criteria. Families were eligible to participate if they meet all the following inclusion criteria:

1. The child’s caregiver(s) gave consent to study participation,
2. The participating child was 10-months-old (± 4 weeks) at the time of baseline assessment,
3. The family spoke Swedish at home,
4. The participating caregiver (mother or father) was able to attend the baseline assessment, the intervention sessions, and the first post-assessment (immediately following the intervention) with their participating child.

Families were not eligible to participate if they meet any of the following exclusion criteria^1^:

1. The child was born prematurely (less than 37 weeks gestations).
2. In the view of parental judgment, the child had an illness or disability that may prevent them from fully participating in the study.

### Intervention

#### Index group

The intervention was a group-based book sharing program for caregivers, “the Mikhulu Trust book sharing programme for children aged 10–24 months” (www.mikhulutrust.org). The program structure and content has been informed by the original Whitehurst model [39, 40]. The program has previously been evaluated with children aged 14–18 months [37, 39, 40]. It was adapted for the targeted age-group by selecting age-appropriate picture-books and by focusing on teaching caregivers’ book sharing techniques most relevant for when sharing books with infants. The intervention program was delivered by a trained facilitator weekly for five consecutive weeks to groups of around 4–6 parent-child dyads. Each session (60 min) was organized around specific topics and incremental techniques; see Table 1 for a description of the content for each session. During each session, the caregivers sat in a semicircle facing the facilitator. Sessions 2 to 5 began with a review wherein the participating caregivers shared and discussed their experiences of book sharing in the preceding week, with the guidance of the facilitator. In each session, key learning points were presented to caregivers through PowerPoint slides. The slides included instructive pictures and video clips to demonstrate the key techniques to apply during book sharing with their child. The caregivers were encouraged to be involved in the session by expressing their opinions and by asking questions about the session content. During this part of the session, toys were available on the floor for the children to explore.

**Table 1:** Intervention sessions

Session	Session content
1	**Introduction and getting started** (using “Vi går på Babyrytmik” by Moa Eriksson Sandberg & Erik Sandberg). The benefits of book sharing to children’s development are presented. The key principles of book sharing are introduced and the caregiver is encouraged to engage the child actively with the book, to facilitate the child’s handling of the book, to help the child to turn pages and steady the book, to follow the child’s cues and interest, to praise the child, to have fun together, and to support their child. The importance of establishing a book sharing routine is emphasized.
2	**Engaging your child and supporting their language** (using “Trycka knappen & Bilen säger brum” by Lotta Olsson & Charlotte Ramel). The caregiver is encouraged to point and name objects, characters and actions in the child’s visual field, indexed by the child’s looking, banging, scratching or pointing; the caregiver is encouraged to respond by naming the object. The caregiver is encouraged to engage their child in the book by using a lively voice and by responding in a positive way to the child’s attempt to communicate. The caregiver is encouraged to repeat words and to make links between the book’s content and “the here and now” by using actions and enacting (e.g., moving their hand up and down to animate the banging on a drum) to facilitate learning.
3	**Linking, enacting, inviting and building** (using “Var är Babbas saker?” by Anneli Tisell & Iréne Johansson). The caregiver is encouraged to use “where” style questions for words that the child understands and to use “what” and “who” style questions for words the child can say (e.g., “Where is the dog?”, “What is this?”). The caregiver is encouraged to enrich the content of the pictures in the book based on what the child knows (e.g., elaborate what is on the page to the child’s wider experiences, such as the picture cat is “just like our neighbor’s cat”).
4	**Making links to everyday life, talking about feelings** (using “Vem är arg?” by Stina Wirsén). The caregiver is encouraged to identify characters’ feelings by pointing and naming facial expressions, by asking “wh-” style questions in relation to emotions, and to talk about the book characters feelings. The caregiver is encouraged to make links between the emotional content in the book and the child’s experiences in everyday life.
5	**Key points and going forward** (using “Katt kan hela dagen” by Sanna Töringe & Kristina Digman). In this session, the key points of the book sharing program were summarized. The facilitator and participants discussed how to continue with book sharing and maintain a routine and how to use book sharing techniques (e.g., following the child’s lead, pointing and naming, engaging the child) during play.

At the end of the PowerPoint presentation, caregivers were presented with the “picture book of the week” and the caregivers and facilitator discuss how they could use the presented book sharing techniques with the specific book. This was followed by an approximately 10 min period in which the caregivers shared the picture book with their child and tried to implement the book sharing techniques under the guidance of the facilitator. All toys are removed during this part to not distract the children. The caregivers keep the “picture book of the week” and were encouraged to use the trained book sharing techniques with their child at home for *about 10 minutes per day*. Together with the “picture book of the week,” the caregivers were given a laminated card with a summary of the key points reviewed in the session.

Around the time when the child participant turned 15 months of age (i.e., 3 months prior to the first follow-up assessment at 18 months of age), the participating families in the index group received a new picture book by mail together with a review of key points from the intervention program and a brief summary of the study’s progress.

For the current trial, the intervention was delivered by a facilitator trained to deliver the intervention, either during a 3-day workshop ran by an accredited trainer of the Mikhulu Child Development Trust (www.mikhulutrust.org) or by a facilitator who received the same training by a trained facilitator (LF).^2^ The facilitator was provided with weekly supervision by LF (an accredited facilitator). During the weekly meetings, the facilitator described the progress of the last week’s intervention sessions, discussed potential challenges (e.g., if participants experienced difficulties in implementing the trained techniques) and practical issues, and reviewed the attendance record.

#### Active control group

The active control group sessions were designed to mimic the sessions of the index group in important ways. The purpose of the active control group was to ensure that any potential intervention effects related to book sharing training were not due to being part of a professionally managed social group or because of receiving more professional attention from researchers. The participants (parent-child dyads) in the active control group attended group sessions for the same amount of time as the index group. They met weekly for five consecutive weeks in groups of around 4–6 parent-child dyads. The group sessions were held at different locations, but each group always met at the same time and at the same location. To control for the potential effect of location, every control group met at the same location as one index group during the same week but at a different time point (e.g., a control group meet in the morning and an index group in the afternoon). The order of the group meetings (index or active control first) was randomized between paired index-control groups prior to the start of the study. The active control group sessions and intervention sessions were held by the same facilitator.

Similar to the index group, during each session, the caregivers sat in a semicircle facing the facilitator. Every control group session was organized around a child development topic: (1) infant active exploration and play, (2) child motor development, (3) child socio-cognitive development, (4) child language development, and (5) child cognitive development. The content of each presentation involved children’s general development and relevant research findings, but it did not include any guidance for parental behavior. The facilitator presented the week’s topic using PowerPoint slides, which included pictures and video-clips. The caregivers were encouraged to be involved in the session by expressing their opinions and by asking questions about the session content. During this part of the session, toys were available on the floor for the children to explore. Prior to the PowerPoint presentation, sessions 2 to 5 began with a review of the last week’s topics and the facilitator encouraged the participating caregivers to share and discuss their child’s development (e.g., in relation to the previous week’s topic or if they had noticed something new in their child’s development). Following the facilitator’s PowerPoint presentation (≈ 30 min), the participants were presented with a large variety of age-appropriate toys (e.g., balls, blocks, hand-puppets, toys with wheels) and were encouraged to play together on the floor. In lieu of a picture book, the participants in the active control group received a gift card of (€10) at each group session.

Around the time when the child participant turned 15 months of age (i.e., 3-months prior to the first follow-up assessment at 18 months of age), the participating families in the active control group received a small toy by mail together a brief summary of the study’s progress.

Prior to the start of the active control group sessions, the facilitator received training in how to deliver the group sessions. The facilitator was also provided with weekly supervision by LF, similar to the supervision of the index group sessions. During the weekly meetings, the facilitator described the development of the last week’s group session, potential challenges (e.g., difficulties in involving participants in discussions, practical issues), and the attendance record was reviewed.

### Outcomes

Outcome data are collected through the use of (1) direct child assessments with eye tracking (Tobii X3-120, Tobii technology, Stockholm), Electroencephalography system (EEG; 128 channel net, Electrical Geodesics Inc., EGI), video-recorded observations, and a tablet test, (2) video-recorded structured observations of parent-child interaction, and (3) parental questionnaires. Study outcomes are presented in Table 2.

**Table 2:** Study outcomes and measures

Outcomes	Measures	Method	Baseline assessment (T1)	Intervention	Post-assessment (T2)	First follow-up (T3)	Second follow-up (T4)
***Primary outcomes***							
Child language	Receptive	Eye tracking: *preferential looking task*	x		x	x	
		Eye racking: *pupil dilation task*	x		x		
		EEG: *N400*	x		x		
		Parent report: *CDI*	x		x	x	x
	Expressive	Parent report: *CDI*	x		x	x	x
Child socio-cognition (joint attention)	Initiating joint attention	Observation: *ESCS*	x		x	x	
	Responding to joint attention	Observation: *ESCS*	x		x	x	
***Secondary outcomes***							
Child executive function		Parent report: *EEFQ*	x		x	x	x
	Simple inhibition	Observation: *“prohibition task”*				x	
	Inhibitory control	Tablet task: *ECITT*				x	
	Working memory	Observation: *“hide and seek task”*				x	
	Shifting	Observation: *reversed categorization*				x	
Parental behavior	Sensitivity and reciprocity	Observation: *BSSI*	x		x		
	Cognitive scaffolding	Observation: *BSSI*	x		x		
	Cognitive scaffolding	Observation: *“problem solving task”*	x		x		
***Moderators***							
Parental socio-economic status	Parental level of education	Parent report	x				
Home literacy environment	Reading habits	Parent report	x		x	x	x
Parental intervention compliance	Attendance record, intervention dose (amount of practice), intervention experience	Facilitator/parent report		x			

*Abbreviations*: *EEG* Electroencephalography, *CDI* Communication Development Inventory, *ESCS* Early Social Communication Scale, *EEFQ* Early Executive Functions Questionnaire, *ECITT* the Early Childhood Inhibitory Touchscreen Task, *BSSI* the Book Sharing Scale for Infants

### Primary outcome measures

#### Child language

Parental reports on a short-version of the *Swedish Early Communication Development Inventory* (CDI) [41] are used at all assessments for assessing *word production and word comprehension* (i.e., expressive and receptive language). From this 90-item checklist, we calculate aggregated scores for expressive and receptive language and used as outcome variables.

*An eye tracking word-picture matching paradigm* is used for assessing *word comprehension* (i.e., *receptive language*) based on the child’s preferential looking pattern [42, 43] at baseline, post-assessment, and first follow-up. This is a screen-based assessment that comprises 32 test trials (i.e., paired-picture trials). All pictures consist of color images of objects representing common nouns (e.g., mouth, bottle, foot). The pictures are presented on a 22″ screen and the participant’s gaze is recorded at 60 Hz with a Tobii X3-60 eye tracking camera (Tobii Technology AB, Stockholm, Sweden). On each test trial, the participant is presented with a pair of pictures for 2500 ms, while hearing a prerecorded sentence that directed them to look at one of the pictures (object/noun). For each trial, we calculate the participants dwell time to the target object (picture) and the distractor object (picture) during an analysis window of 367–2500 ms. We then calculate the proportion of target looking time by subtracting the time the child looked at a picture when it was a distractor from the time when it was a target. Proportion of target looking time was averaged across trials and used as the outcome measure.

*An EEG and eye tracking based mismatch-paradigm* [44] is used to assess child *word comprehension* at baseline and post-assessment. The child participants sit in front of 22″ monitor, with a Tobii X3-60 eye tracker attached to its bottom, while wearing a 128-channel Geodesics Sensor Nets. This task consists of 48 trials. Every trial starts out with displaying a scene consisting of a floor and a blue occluder on the monitor. A sentence with a word (“Look at X”) was presented over headphones to the caregiver who has been instructed to repeat the sentence to their child. Once the sentence is uttered, the experimenter pushes a button. Following this, the occluder disappear and an object (representing a common noun, e.g., a duck, a ball, or a car) is revealed. Objects are congruent (50%) or incongruent (50%) with the word spoken by the caregiver. This task has two outcome measures: (1) we measured an event-related brain potential known as the N400 (based on differential amplitude on congruent and incongruent trials, averaged across trials), and (2) we measure a pupil dilation difference score based on differential dilation on congruent and incongruent trials, baseline corrected and averaged across trials (see for example [45]). For both measures, the assumption is that if the child has developed a word knowledge of a specific word/object, they will detect the mismatch between presented word and object on incongruent trials. This mismatch is then reflected in enhanced N400 amplitude and increased pupil dilation on incongruent trials (vs congruent trials).

#### Child joint attention

The child’s ability to *initiate and respond to social communication*, that is, *joint attention skills*, is assessed with two tasks, the *Object Spectacle* Task and the *Gaze Following Task*, from the Early Social Communication Scales (ESCS) [46]. This assessment is made at the baseline, post-assessment and at the first follow-up assessment. In both tasks, the experimenter and the child are sitting at a table facing each other.

In the *Object Spectacle* Task, the experimenter activates a toy for 6 s in front of the child, but out of the child’s reach. This procedure is repeated for a total of 9 trials using three different toys (a wagging dog, a hand puppet, and a rattle). In each trial, we measure the child’s number of alternating gazes between the toy and the experimenter based on video records. The outcome measure is the proportion of alternating gazes across trials.

The *Gaze Following Task* involves a sequence of the experimenter pointing and gazing at four posters hanging on the wall. The four posters are located to the left, right, left behind, and right behind the child. The child is presented with a total of 8 trials (i.e., every poster is the target on two occasions). On all trials, the experimenter captures the child’s attention and then turns to point and gaze toward a poster. Based on video records, we calculate the proportion of correct responses (i.e., gaze following) and use this as our outcome measure.

### Secondary outcome measure

#### Child executive function

Child EF is assessed at all four assessments using the Early Executive Functions Questionnaire (EEFQ) [47]. This is a 31-item questionnaire (using a 7-point scale) consisting of four sub-scales: *Inhibitory Control*, *Working Memory*, *Flexibility*, and *Regulation*. We will use the mean scores from this questionnaire as outcome measures.

Child EF is also assessed at the first follow-up assessment with four different observational lab tasks targeting *simple inhibition*, *inhibitory control*, *working memory* and *shifting (or cognitive flexibility)*. *Simple inhibition* is measured by the “Prohibition Task” [48]. The child and the experimenter sit at a table facing each other. The experimenter presents an attractive toy to the child. While having eye contact with the child, the experimenter shakes their head and says: “now, (“child’s name”), you are *not* allowed to touch this” while simultaneously placing the toy on the table within the child’s reach. The outcome variable is the latency to touching the toy, with a maximum of 30 s, and will be coded from video records. A longer waiting time indicates higher inhibition.

*Inhibitory control* was assessed with the Early Childhood Inhibitory Touchscreen Task (ECITT) [49]. During this task, the child sits next to the parent. The experimenter holds an iPad in front of the child. Following a warm-up game to get familiarized with the tablet and one warm-up trial, two buttons, one at the left and one at the right, are presented on the screen and the child is instructed to press the button with the happy face. If the child presses the correct button, an animation is shown. If the child presses the incorrect button, no animation is shown. The happy face appears in the same (prepotent) location on 24 trials (75% of trials) and in the inhibitory location on 8 trials (25% of trials). The outcome measures are reaction time (i.e., time between presentation of the buttons and the child’s button pressing) and mean accuracy (i.e., percentage correct in prepotent trials minus the percentage correct in inhibitory trials). A larger score indicates lower inhibitory control.

*Working memory* is assessed with a hide-and-seek task [50]. The hiding locations are colored drawers, part of a small table chest. The tasks consist of two warm-up trials and four test trials. The child and the experimenter are sitting at a table. On each trial, the experimenter hides the toy in one of the drawers while saying “Now I am hiding it here” and then covers the chest with a cloth. After 5 s, the child is encouraged to search for the toy. The child receives a score of 4, 3, 2, or 1 according to whether they were successful on the first, second, third, or fourth attempt, respectively. Children who did not succeed after four attempts are given a score of 0. The mean score over all test trials was calculated and used as the outcome measure. This score was coded from video records. A larger score indicates stronger working memory.

*Shifting* is assessed with the task Reversed Categorization [51]. The child and experimenter were sitting at a table. Children are asked to sort red and yellow blocks into a red and yellow bucket according to their color and then to reverse this categorization so that yellow blocks would go to the red bucket and vice versa. We calculate proportion of correctly sorted blocks on the categorization trials (according to color; raw score = 0–6) and the reversed trials (raw score = 0–12). The outcome measures were coded from video records. A larger score indicates stronger shifting abilities.

#### Parental scaffolding, sensitivity, and reciprocity

Parental cognitive scaffolding, sensitivity and reciprocity are being assessed by structured observations of parent-child interactions in a book sharing and a problem-solving task. The observations will be coded from video records using the Book Sharing Scale for Infants (BSSI) [52] for the book sharing task, whereas the coding of the problem-solving task is based on the Autonomy Support Scale [53].

### Potential moderators

The following variables will be used as potential moderators: *family socio-economic status* (indexed at baseline by parental report on highest parental education level: elementary school, high school, university < 3 years, or university >3 years ), *home literacy environment* (indexed by parental report on reading habits at all four assessments, e.g., frequency of reading for the child: never, 1–2 times per month, 1–2 times per week, or everyday/almost every day), and *parental intervention compliance* (indexed by intervention sessions attendance records, and self-reported practice of shared-reading between session, reported as days per week and minutes per day, and intervention experience, e.g., whether they think the book sharing training has been beneficial to them).

### Other measures

The study will also include questionnaire items on demographic information. For example, information on parental age and gender, first language, child monolingualism status, and gender. The participants will additionally be assessed with the Bayley Cognitive and Fine Motor Skills Scale (Bayley Scales of Infant and Toddler Development-III] [54]. This data will be used for descriptive purposes and for examining whether demographic characteristics and the Bayley-III outcome measures are balanced between the index and control group at baseline.

### Sample size

The originally targeted sample size was 140 children (10-month-old ± 4 weeks) and their caregivers (70 dyads in each arm). The sample size was calculated on the basis of the primary outcome measure language. The power calculation was conducted in G*Power [55] using 80% power and alpha set to = .05. The effect size (*d*) for the power calculation was based on the results reported in a recent meta-analysis by Dowdall et al. [26]. The meta-analysis [26] was based on RCT book sharing interventions targeting young children (1 to 6 years) and reported an overall effect size of *d* = .54 for expressive language in studies using medium to high intensity intervention dose (i.e., more than 60 min contact time). Based on this estimate, an index and control sample of 55 dyads in each arm would therefore be sufficient to detect a difference (two-tailed) and would allow for an attrition rate > 10%. The study’s final sample size was 115 parent-child dyads (index group, *n* = 59; active control group, *n* = 56). Thus, the study’s sample size is sufficiently powered.

### Recruitment

Recruitment began in January 2020 and ended in August 2021.^3^ The first recruitment wave took place in January to February 2020, the second recruitment wave took place between May and October 2020, and the third recruitment wave took place between July and August 2021. Participants were recruited to the study using several methods. A part of the sample was recruited from the BASIC project—a large epidemiology study investigating maternal health during and after pregnancy [56]. Mothers participating in the BASIC-project was asked if they were interested in being contacted by the Uppsala Child and Baby Lab about a study on child development. Recruitment also took place by contacting families with a general interest in participating in child developmental studies at the Uppsala Child and Baby Lab. Other means of recruiting potential participants included sending letters describing the study to families in Uppsala municipality, using advertisement for the study on Facebook, and by putting up flyers and verbally informing parents visiting “open pre-schools” in Uppsala. All potential participants, i.e., caregivers with a child of appropriate age who expressed interest in participating in the study, were provided with an invitational package that described the study and included copies of the consent form. Caregivers who expressed an interest in the study were then screened via telephone for eligibility.

Of 187 dyads assessed for eligibility, 151 met all inclusion criteria and none of the exclusion criteria and gave consent to participate in the study. They were then randomized to an index group or active control group. Of these, 115 completed the baseline assessment, 109 completed the post-assessment, and 98 completed the first follow-up assessment at 18 months. The follow-up assessment at 24 months is still ongoing at the current date.

### Assignment of interventions: allocation

#### Sequence generation

A computer-generated randomization sequence (http://www.randomization.com) was used to allocate participants in a 1:1 ratio to the intervention group and the active control group. Blocked randomization with a 1:1 ratio was used to ensure that the study groups were of approximately the same size.

#### Concealment mechanism

Allocation concealment was released to the participants after being recruited into the study and giving consent to being part of the study prior to baseline assessment. The allocation of participants to the intervention group or the active control group was done by a person on the research team who was not involved in participant assessments.

#### Blinding (masking)

Due to the nature of the intervention and for ethical reasons, the intervention facilitator and the participants are not blinded to group allocations. They are aware of the existence of two groups (i.e., a reading group and a play group), but not of the study hypothesis. All assessments of participating caregivers and children are conducted by a data collector, who is blind to group belongings. Participating parents are asked to not reveal their group belonging to the data collector. All coders of video data will be blinded to group allocation. Allocation assignment will be concealed to the researchers at the analysis stage.

### Data collection

#### Data collector training

A team of data collectors have been trained in lab assessments and administration of the questionnaires. All data collectors had a BA or higher degree in psychology (or equivalent subject) and were trained in the assessments prior to data collection (e.g., by running pilot assessments), including consent and referral procedures. The training and administration of tasks followed a data collector manual developed by LF and JG. To monitor and ensure the fidelity of assessment administration, LF and JG made regular in vivo checks with data collectors and through examination of video records.

### Coding and reliability

Videos of caregiver-child interactions and child joint attention and EF tasks are being coded by independent trained coders who are unaware of participant’s intervention allocation. Reliability will be established from randomly selected 20% of video records per task.

#### Procedure

Data collection occurs at four time points: at baseline, following the 5-week intervention, and 6 and 12-months post intervention. At each time point, the participating caregiver complete a questionnaire using a secure online platform (https://sv.surveymonkey.com). Lab assessments take place at the first three assessment time points. Prior to each lab assessment, the child’s caregivers (legal guardians—when applicable) receive written information about the study purpose and procedures. Before each study visit, we collect written consent from the child’s caregivers. All participants are informed that that they have been given an anonymous identity number and that their data will be treated confidentially, that published reports of results from the study will be reported at a group level (meaning that it will not be possible to identify individuals), and that they can stop participating in the study at any time without giving any reasons. Participants are also informed that they have the right to have their data removed from the database at any time. During each study visit, the participating child’s caregiver is with the child at all time and can pause or stop data collection at any time. Similarly, the data collector pauses or stops the data collection during study visits if the child participant shows signs of discomfort or tiredness. Lab assessments last for up to 1.5 h, including breaks. The participants receive a gift card (€10) at each lab visit as a form of travel reimbursement.

### Criteria for discontinuing or modifying allocated interventions

Intervention session will be discontinued if participants request to withdraw from the study.

### Provision for post-trial care

Participants were informed about the risk associated with participating in the trial. Referrals were made if deemed appropriate and necessary. There were no plans for post-trial care.

#### Retention

Structures are in place to maximize participant retention. This includes making efforts to accommodate the participating caregiver and child when it comes to scheduling and re-scheduling of assessments, sending e-mail and text message reminders about upcoming scheduled assessment, group sessions and questionnaires, and sending information letters with updates on study progress to participating families.

### Data management

All data, including eye tracking data, EEG data, questionnaire data, and video records of observational task are submitted to a secure university server. All data are stored and managed according to current regulation on personal data management. Participants’ consent forms are stored in a locked storage area that is separate from the data obtained. All research data are “coded” and identified only by participant identification numbers to protect the participants identity and maintain confidentiality.

### Statistical methods

All statistical analysis will be conducted using the SPSS and R software. Data will be screened to check for data entry errors and to test if the data meet the assumptions of parametric procedures. The pattern and amount of missing data will be examined and handled by using maximum-likelihood estimates (or similar estimates), if appropriate. Baseline data and demographic characteristics will be described using summary statistics (means and standard deviations or number and percentages). Group baseline differences will be assessed using independent samples *t* test, chi-squared test, and Mann-Whitney *U* test. Fidelity in terms of adherence and the dose of the intervention will be summarized using descriptive statistics. All tests will be two-sided and 5% will be used as the level of significance. Effect size measures (e.g., Cohen’s *d*) will be calculated to describe intervention effects.

The statistical analyses of the primary outcomes will be performed blinded to treatment allocation. In accordance with CONSORT guidelines, intention-to-treat principles will be used to prevent systematic bias. Linear mixed models or analysis of covariance will be used to assess intervention effects at post intervention and at the follow-ups. Baseline scores will we controlled for as covariates. Other potential covariates (e.g., child monolingual status) will be investigated by examining correlations between covariates and the outcome variables.

#### Mediator analysis

We will conduct mediator analysis to investigate potential mechanisms of change following the intervention. Specifically, we will examine if improvements in parental scaffolding, sensitivity, and reciprocity mediates improvements in child language, joint attention, and EF at post interventions and follow-ups.

#### Moderator analysis

In addition, moderator analysis will be conducted to examine whether specific groups benefit more or less from the intervention. Specifically, we will investigate the associations between improvement status and participants’ characteristics, such as family socio-economic status, quality of home literacy environment, and parental intervention fidelity (e.g., number of sessions attended).

### Dissemination plans

We will disseminate the study findings in several ways. We will publish them in highly ranked international peer-reviewed journals. Results will also be orally presented at international conferences on child development, psychology, and education. We will communicate our results to the wider public via local and national media, by using social media (Facebook and Twitter) and by posting summaries of the study at Uppsala Child and Baby Lab University webpage.

## Discussion

The REaL trial is an evaluation of a book sharing intervention for caregivers and their 10-month-old children. The intervention is conducted over 5 weeks and is designed to evaluate intervention effects on child language, joint attention, and EF. Its impact on parental behaviors (cognitive scaffolding, sensitivity, and reciprocity) will also be assessed. The trial includes long-term follow-ups and will therefore be able to clarify the durability of the intervention effects and potential spill-over effects. The intervention is relatively brief and involves modest levels of training. Thus, demonstrations of positive intervention effects on child and parental outcomes would be of significance for the development of early interventions to promote child development.

## Trial status

Recruitment to the trial began in January 2020. Due to the COVID-19 pandemic, recruitment paused between February 18 to May 15, 2020, and between October 21, 2020, and July 7, 2021. At the point of submitting this manuscript to the journal (17 March 2021), 97 out of the final 115 participants in the sample had been recruited. Recruitment to the study ended 5 August 2021. This paper represents version 1 of the protocol.

## Supplementary Information

**Additional file 1.** SPIRIT 2013 Checklist: Recommended items to address in clinical trial protocol and related documents.

## Data Availability

Authorship will be granted for substantive contribution to the design, conduct, interpretation, and reporting of the trial. Publications will be made open access. Datasets generated during the current study will be deposited in publicly available repositories (where available and appropriate) 1 year after the completion of the study.

## Abbreviations

BSSI: Book Sharing Scale for Infants
CDI: Communication Development Inventory
ECITT: Early Childhood Inhibitory Touchscreen Task
EEG: Electroencephalography
EEFQ: Early Executive Functions Questionnaire
EF: Executive function
ESCS: Early Social Communications Scales
RCT: Randomized controlled trial
REaL: Roadmap to Executive functions and Language

## Footnotes

1 Due to the COVID-19 pandemic, recruitment paused after the recruitment of 38 participants. When recruitment was reuptaken, the following exclusion criteria were added: (1) someone in the family belongs to a risk-group in relation to COVID-19, or (2) the participating caregiver has a work that is essential for society.

2 Following agreement from Peter Cooper, one of the founders of Mikhulu trust and the specific book -sharing program.

3 Recruitment was initially planned to take place between January to August 2020. Due to the COVID-19 pandemic, including national and local restrictions, recruitment of participants paused between mid-February to end of May 2020, and again from mid-October 2020 to beginning of July 7, 2021.
